# Septal myectomy with mitral valve surgery in patients after alcohol septal ablation

**DOI:** 10.1093/icvts/ivac010

**Published:** 2022-02-01

**Authors:** Kostiantyn V Rudenko, Vasyl V Lazoryshynets, Lidiia O Nevmerzhytska, Mariia O Tregubova, Polina A Danchenko

**Affiliations:** 1 Department of Myocardial Pathology, Heart Transplantation and Mechanical Circulatory Support, Amosov National Institute of Cardiovascular Surgery NAMS of Ukraine, Kyiv, Ukraine; 2 Department of Surgical Treatment of Congenital Heart Diseases in Infants, Amosov National Institute of Cardiovascular Surgery NAMS of Ukraine, Kyiv, Ukraine; 3 Department of Radiology, Amosov National Institute of Cardiovascular Surgery NAMS of Ukraine, Kyiv, Ukraine; 4 Department of Surgery with Course of Emergency and Vascular Surgery, Bogomolets National Medical University, Kyiv, Ukraine

**Keywords:** Hypertrophic obstructive cardiomyopathy, Alcohol septal ablation, Mitral valve repair

## Abstract

**OBJECTIVES:**

We studied 16 patients after failed alcohol septal ablation who underwent extended septal myectomy to analyse the results of surgical correction and identify technical pitfalls the surgeons may be confronted by.

**METHODS:**

Between October 2017 and March 2019, 16 patients underwent surgical extended septal myectomy with accompanying anomalous secondary chordae resection, papillary muscles mobilization [in 9 (56.3%) patients], and anterior mitral leaflet plication after previously failed alcohol septal ablation. Routine preoperative computed tomography or cardiac magnetic resonance planning and intraoperative transoesophageal echocardiography were performed in each of the studied patients. Major technical features were identified and complemented during septal myectomy of the calcified interventricular septum.

**RESULTS:**

The mean age of the studied patients accounted 50.5 ± 14.6, median—54; males—5 (31.3%). Mean cross-clamp time accounted 52 ± 7.2 min. Calcified basal interventricular septum was identified in 2 (12.5%) patients. No iatrogenic ventricular septal defect (0%) was made during surgical correction. Peak systolic pressure gradient decreased from 86 (interquartile range: 75–104.7) to 20 (16–22) mmHg (*P*< 0.001). No patients with moderate or severe mitral regurgitation were identified, whereas before the procedure, the number of those accounted 13 (81.2%) individuals. In-hospital and overall mortality after septal myectomy accounted 0%.

**CONCLUSIONS:**

Extended septal myectomy in patients who previously underwent alcohol septal ablation is a safe procedure that affects all pathological manifestations of the disease. Routine preoperative computed tomography or cardiac magnetic resonance provides detailed anatomy of the anomalous left ventricle and subvalvular structures and allows to measure the extension of myectomy preventing the occurrence of iatrogenic ventricular septal defect. Septal myectomy of the calcified interventricular septum requires avoidance of ‘one-piece technique’ since fragmental myectomy allows visually control the adequacy of the left ventricle outflow tract release.

**Figure:**
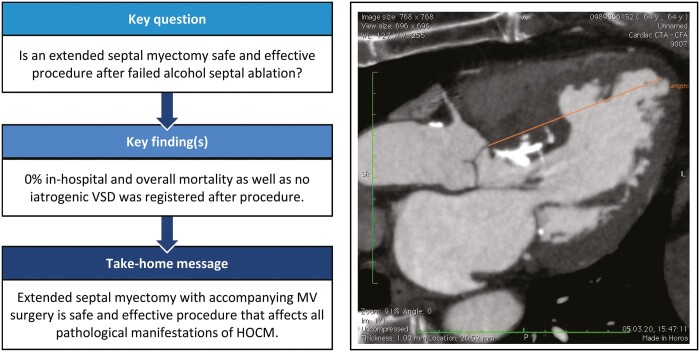

## INTRODUCTION

For more than 50 years, transaortic septal myectomy is known to be the gold standard for treatment of hypertrophic obstructive cardiomyopathy (HOCM) [1, 2]. This safe and effective surgical procedure [3–5] allows to affect all pathophysiological mechanisms of the genetically inherited disease. In addition, relief of the obstruction during septal myectomy with concomitant mitral valve (MV) surgery restores the functional capacity and normal life span, and the survival rate of surgical patients is similar to that of the general matched population [6].

With the development of era of catheter-based treatment, septal myectomy has been dramatically eclipsed by less invasive approach—alcohol septal ablation (ASA). Although ASA is performed percutaneously providing better cosmetic effect, the procedural risks of death and morbidity are not lower than the risks of operation [7], and the risk of reintervention after ASA is reported to be much higher compared to septal myectomy [8].

Taking into account that the mechanisms which predispose the left ventricular outflow tract (LVOT) obstruction involve not only basal septal hypertrophy, small size of the left ventricle (LV), hypercontractility of the LV [9], but also the anomalies of the subvalvular apparatus of the MV [10], choosing the less invasive approach for these patients may not always correlate with adequate postoperative haemodynamic result. It has been reported that overall haemodynamic success of the ASA is about 70%, with an approximate 20% incidence of recurrence of severe symptoms [11]. Additionally, the myocardial infarction created by injection of ethanol is associated with higher incidence of early complications compared to surgical correction [8, 12, 13].

In this study, we present the results of treatment of 16 patients with HOCM who underwent surgical septal myectomy with accompanying MV surgery after failed ASA and sought to determine the technical pitfalls the surgeons may be confronted by during the procedure.

## MATERIALS AND METHODS

### Study population

We studied 16 patients presented at our Institute between October 2017 and March 2019 who previously underwent ASA and had significant residual symptoms of progressive heart failure. All patients were referred to surgical treatment that involved 4 steps of surgical correction: extended/shallow septal myectomy, secondary chordae cutting, papillary muscles (PM) mobilization and anterior mitral leaflet (AML) plication. Three patients underwent ASA at our Institution and 13 patients underwent ablation at other medical facilities. One patient underwent ASA, dual-chamber pacemaker and implantable cardioverter-defibrillator (ICD) implantation. Three patients previously underwent more than 1 ASA procedures.

#### Ethical statement

This study had been approved by Amosov National Institute of Cardiovascular Surgery Review Board and Ethics Committee (protocol № 6074391 from 12 November 2020) and informed written consent had been acquired from each patient included to the study.

#### Data availability statement

The data that support the findings in this study are available on request from the corresponding author.

### Diagnosis

Following the latest recommendations [1, 2], the diagnosis of HOCM in each patient was established based on the presence of hypertrophied myocardium with non-dilated LV in the absence of other cardiac or systemic diseases which could have explained the hypertrophy. The indications for surgery involved the systolic pressure gradient (SPG) ≥50 mmHg and symptoms refractory to adequate medical treatment. All 16 patients underwent baseline echocardiographic and imaging examinations including transthoracic echocardiography and computed tomography (CT) of the heart or cardiac magnetic resonance (CMR) with late gadolinium enhancement with subsequent calculations of the size of the right ventricle, LV and interventricular septum (IVS) to detect structural abnormalities, degree of fibrotic process and congenital heart defects.

### Preoperative planning

**Figure 1:**
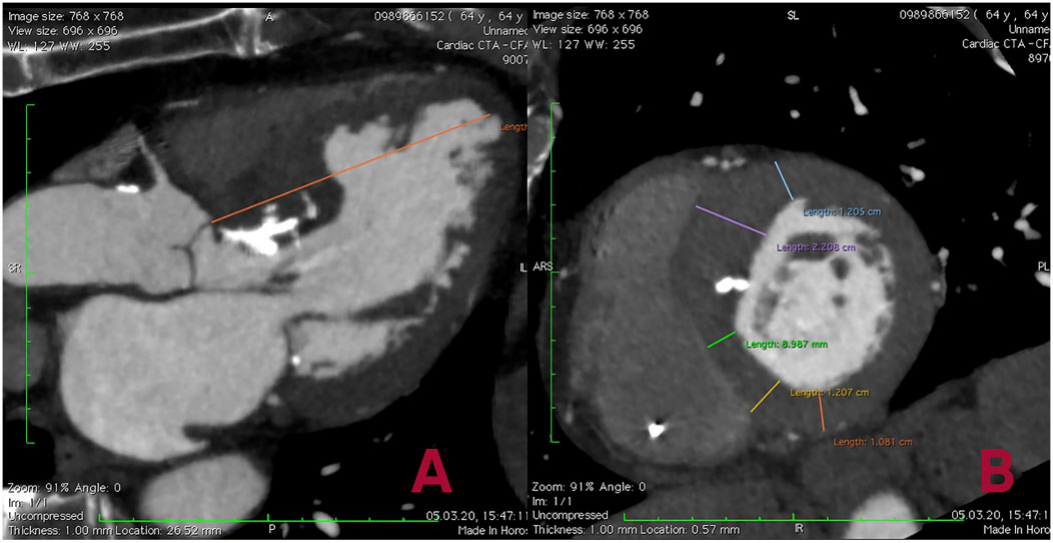
Preoperative computed tomography planning using long- (**A**) and short-axis (**B**) views, where the distance from the aortic valve ring to the left ventricular apex in the 3-chamber view and the thickness of the left ventricle and interventricular septum are measured at a distance of 8 mm from the aortic valve ring at the end of diastole.

During CT in each patient, the thickness of the anterior, anteroseptal, posteroseptal and posterior segments of the LV was measured in a 2-chamber short-axis view (Fig. 1). The starting point from which the measurement of the thickness of the above-mentioned segments was performed, was determined in a 3-chamber long-axis view at a distance of 8 mm from the aortic valve (AV) ring. Subsequent measurements were performed at every 8 mm beginning from the basal segments of the LV to the PM level (equatorial zone of the LV) to reconstruct the distribution of hypertrophy at the different levels of the LV. The length of the LV was measured as the distance from the AV ring to the LV apex in a 3-chamber long-axis view. The estimated length of resection during septal myectomy was defined as the sum of consecutive hypertrophied LV segments measured at every 8 mm level beginning from the AV ring. During surgery, data obtained from the detailed CT-analysis of IVS thickness were used as a guide for septal myectomy and allowed to avoid iatrogenic ventricular septal defect (VSD) during procedure.

### Transoesophageal echocardiography

**Figure 2:**
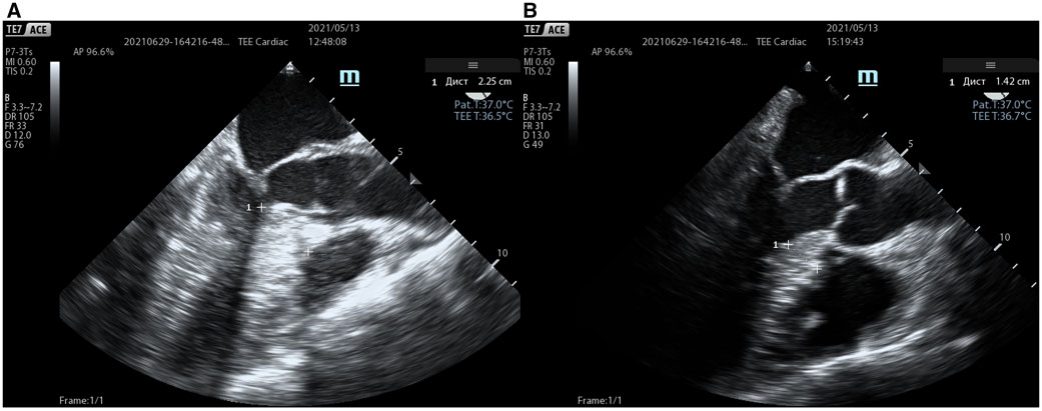
Intraoperative TEE images (mid-oesophageal aortic valve long-axis view) in early systole before (**A**) and after (**B**) procedure; elimination of the left ventricular outflow tract obstruction with concomitant procedures on the mitral valve subvalvular apparatus. Septal thickness (1) was measured before and after correction. TEE: transoesophageal echocardiography.

After induction of anaesthesia, intraoperative transoesophageal echocardiography (TEE) was performed to determine the extent of myectomy, as well as to assess the morphology of the MV and the presence of associated primary MV anomalies (Fig. 2). TEE was repeated in the operating room immediately after stopping the cardiopulmonary bypass for the assessment of residual gradient on the LVOT and regurgitation on aortic and MVs, as well as for the detection of possible surgical complications (e.g. iatrogenic VSD). In addition, TEE was used to measure the coaptation depth in mid-oesophageal 4-chamber view and coaptation-to-septal distance in mid-oesophageal AV long-axis view before and after procedure as one of the key predictors of residual systolic anterior motion (SAM) and mitral regurgitation (MR). Coaptation depth was defined as the shortest distance between the coaptation and the annular plane, while coaptation-to-septal distance was defined as the shortest distance between the coaptation point and the IVS at early systole.

### Surgical procedure

Surgical correction included a series of steps: extended myectomy, resection of fibrotic and retracted secondary chordae of the MV, PM mobilization and, in case of necessity, AML plication with reduction of its size and area.

**Figure 3:**
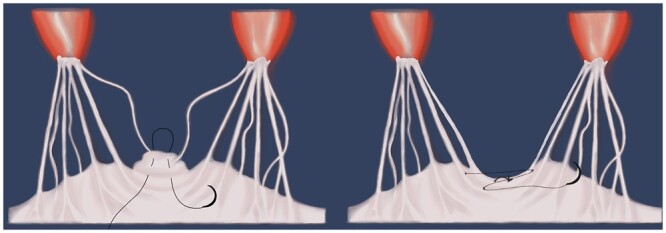
Plication of the anterior mitral leaflet technique. Placing a vertical everting U-suture at the edge of the leaflet in the zone of excess tissue (**A**). Then, an additional z-suture was placed to approach 2 primary chordae (**B**).

Septal myectomy was performed during cardiopulmonary bypass with mild general hypothermia. Using the exposure through the oblique aortotomy the myectomy was begun from 2 longitudinal incisions in the basal part of the IVS, 2–3 mm below the AV, gradually continuing the resection more distally, to the base of the PM (equatorial zone), creating a trapezoidal muscular band which is wider towards the apex than at the subaortic level. Myectomy was always performed using the data obtained from CT or CMR imaging in order to prevent iatrogenic VSD since the thickness of the IVS on various levels was different due to extensive fibrotic changes of the IVS. In case of the calcified IVS, the myectomy was performed fragmentally by resection of small parts of stiff myocardium in order to achieve better visual control of the LVOT release (Fig. 3). After removal of the cardiac muscle, the cavity of the LV was flushed several times with normal saline in order to remove any small particles of the calcified IVS from the heart and prevent the embolic events.

After septal myectomy, an intervention on the subvalvular mitral apparatus was performed. Fibrous and muscular structures that connect PM with IVS or LV free wall were present practically in all patients with HOCM and were limiting the mobility of the PM. Such structures, which can be identified on CT or, sometimes, only at the time of surgery, were systematically resected in each of the examined patient for the purpose of improvement of the PM mobility (PM mobilization). Fibrotic and retracted chordae (pathological secondary chordae) or fibrous attachments between the AML and PM were found in the majority of patients with HOCM. These structures routinely underwent resection, which decreased the depth of coaptation of the MV leaflets and prevented the phenomenon of SAM in the postoperative period. In case of diastasis between the places of attachment of the primary chordae to the edge of the MV leaflet over 5 mm, this area was plicated. After the area of diastasis and the excess tissue of the AML in between 2 primary chordae was identified, a vertical everting U-suture was placed at the edge of the leaflet. Then, an additional z-suture was placed to approach 2 primary chordae (Fig. 3).

### Follow-up

The follow-up was complete and averaged 36 ± 14 months (maximum of 47 months). Follow-up data were collected from medical charts or written correspondents from referring cardiologists of the patients. Vital status assessment was performed via telephone interview directly with patients or their relatives and confirmed by the absence of death certificated that were requested at the medical facilities. The primary endpoints involved cardiac death, advanced heart failure and ICD discharge at follow-up.

### Statistical analysis

Categorical variables are expressed as frequencies and percentages, and continuous variables are expressed as mean ± standard deviation or median [interquartile range (IQR)]. Fisher’s exact test was used to compare categorical variables and Wilcoxon rank-sum test was used to compare continuous variables. All reported *P*-values are 2-sided. The Kaplan–Meier method was used to draw survival curves and calculate survival statistics for long-term outcomes. SPSS statistical software (IBM^®^ SPSS^®^ Statistics 22.0, Chicago, IL, USA) was used for all the calculations.

## RESULTS

There were a total of 16 patients [5 males (31.3%)] who underwent extended/shallow myectomy accompanied by MV surgery after failed ASA. The age of the patients ranged from 11 to 65 years old, and the mean age was 50.5 ± 14.6 (median—54). Three (18.8%) patients previously underwent more than 1 ASA, and other 2 (12.5%) had right bundle branch block after ASA. CT was performed in 13 (81.25%) patients and CMR in 3 (18.75%) patients. Preoperative baseline characteristics are collected in Table 1.

**Table 1:** Preoperative baseline characteristics of 16 studied patients

Variable	Value
Age, mean ± SD (median)	50.5 ± 14.6 (54)
Males, *n* (%)	5 (31.3)
Paroxysmal/persistent AF	1 (6.3)
RBBB, *n* (%)	2 (12.5)
Preoperative infective endocarditis	1 (6.3)
SCD risk (calculated in 15 patients)	
Mild, *n* (%)	6 (40)
Moderate, *n* (%)	7 (46.7)
High, *n* (%)	2 (13.3)
Preoperative ICD implantation	1 (6.3)
Preoperative pacemaker implantation	1 (6.3)
Family history of SCD	4 (25)

AF: atrial fibrillation; ICD: implantable cardioverter-defibrillator; RBBB: right bundle branch block; SCD: sudden cardiac death; SD: standard deviation.

### Procedural features

The average cross-clamp time was 41.5 ± 3.6 min. Fourteen (87.5%) patients underwent extended septal myectomy and 2 patients (12.5%)—shallow myectomy. Calcified IVS was observed in 12.5% (2) of all the cases and was excised by fragments, providing adequate visual control of the amount of myocardium needed to be removed in order to release the LVOT. In other 14 (87.5%) patients ‘one-piece myectomy’ technique was applied. The average mass of the resected myocardium accounted 5.2 ± 0.2 g. Resection of the pathological secondary chordae was performed in all 16 (100%) studied individuals, while the AML was plicated in 9 (56.3%). The procedure of PM mobilization was performed as follows: the anterior group of PM was mobilized in 3 (18.8%) patients, posterior group—in 1 (6.3%) patient, and both groups were mobilized in 6 patients (3.75%). One (6.3%) patient had a duplication of the anterior group of PM and a fibrotic bundle attaching to A1 segment of the AML which was resected during surgery. One (6.3%) patient underwent MV replacement with bileaflet mechanical prosthesis (St. Jude Medical™, Inc. №29) due to existing preoperative infective endocarditis.

Intraoperatively, coaptation depth and coaptation-to-septal distance were measured using TEE at early systole. According to the data revealed, coaptation depth after MV repair accounted 5.4 ± 1.3 mm vs 9.6 ± 3.1 mm before the surgery (*P* < 0.001). Coaptation-to-septal distance prior correction was 1.77 ± 0.81 mm, whereas after the procedure it accounted 3.32 ± 0.78 mm (*P* < 0.001).

No iatrogenic VSD was made during septal myectomy. None of the patients had reoperation or re-exploration for bleeding after the surgery.

### Postoperative outcomes

The average in-hospital stay accounted 8.2 ± 2.4 days. All 16 studied patients had significant improvement in their symptoms and haemodynamic parameters after surgery (Table 2). The LVOT release resulted in the dramatic decrease of the SPG from 86 (IQR: 75–104.7) mmHg to 18.8 (IQR: 14.3–26) mmHg at discharge (*P* < 0.001).

**Table 2:** The results of septal myectomy in patients with previous ASA

Variable	Value				
	Before surgery	At discharge	2 years after procedure		*P*-value
NYHA functional class					
I, *n* (%)	1 (6.3)	2 (12.5)	4 (25)		0.038
II, *n* (%)	7 (43.8)	14 (87.5)	12 (75)		0.047
III, *n* (%)	8 (50)	0 (0)	0 (0)		0.019
IV, *n* (%)	0 (0)	0 (0)	0 (0)		<0.001
Postoperative echocardiography data					
Peak SPG on LVOT at rest or on exertion, mmHg; median (IQR)	86 (75–104.7)	18.8 (14.3–26)		20 (16–22)	<0.001
SAM, *n* (%)	15 (93.75)	0 (0)		0 (0)	0.014
Mitral regurgitation degree, *n* (%)					
0	0 (0)	0 (0)		1 (6.3)	<0.001
1	3 (18.8)	15 (93.7)		15 (93.7)	0.016
2	11 (68.8)	1 (6.3)		0 (0)	0.029
3	2 (12.5)	0 (0)		0 (0)	0.011
4	0 (0)	0 (0)		0 (0)	<0.001
LV basal septum, mm; median (IQR)	22.5 (21–26)	14 (13–15.3)		14 (13.3–16)	<0.001
LV posterior wall, mm; median (IQR)	13 (12.3–15)	13 (12.3–14)		13 (12.3–14)	0.083
LA diameter, mm; median (IQR)	35 (42–48.5)	42 (40–46.3)		42 (40–44.8)	<0.001
EDV, ml; median (IQR)	87.5 (75.3–97.5)	91 (87.5–101.5)		93 (88.3–102)	0.016
AML, mm; median (IQR)	38 (36–40.5)	34.8 (32.5–36.3)		33.5 (31–35.8)	<0.001
EF, %; median (IQR)	64.5 (61.3–65)	61.3 (60.5–64.8)		60.5 (58.3–63)	0.032

Given *P*-values are the comparisons between preoperative and late follow-up data.

AML: anterior mitral leaflet; ASA: alcohol septal ablation; EDV: end-diastolic volume; EF: ejection fraction; ICD: implantable cardioverter-defibrillator; LA: left atrium; LV: left ventricle; LVOT: left ventricular outflow tract; NYHA: New York Heart Association; SAM: systolic anterior motion; SCD: sudden cardiac death; SD: standard deviation; SPG: systolic pressure gradient.

**Table 3:** Major complications after surgery

Postoperative LBBB, *n* (%)	11 (68.8)
Complete AV-block, *n* (%)	2 (12,5)
Postoperative AF, *n* (%)	0 (0)
New pacemaker implantation, *n* (%)	2 (12.5)
New ICD implantation, *n* (%)	0 (0)
Sudden cardiac death, *n* (%)	0 (0)
ICD discharge, *n* (%)	1 (6.3)
Advanced heart failure, *n* (%)	0 (0)
Cardiac death, *n* (%)	0 (0)
All-cause death, *n* (%)	0 (0)

AF: atrial fibrillation; AV: aortic valve; ICD: implantable cardioverter-defibrillator; LBBB: left bundle branch block.

In addition, an echocardiographic assessment of the MR was performed and distributed on a scale from 0 to 4 (0—no MR, 1—mild MR, 2—moderate MR, 3—moderate-to-severe MR, 4—severe MR). Each patient had a certain degree of dynamic MR caused by the presence of SAM in 15 (93.75%) of patients. In 1 (6.3%) patient, MR was caused by the presence of the vegetation on the AML which was the indication to MV replacement. In this study, 3 (18.8%) individuals had mild, 11 (68.8%)—moderate, and 2 (12.5%)—moderate-to-severe MR, respectively. No patients with moderate or severe MR were identified after procedure. In addition, the relief of the LVOT and reduction of SAM in all these patients resulted in significant improvement of their New York Heart Association functional class. Four (25%) patients were in New York Heart Association class I, and 12 (75%) patients were in New York Heart Association class II after surgical intervention.

**Figure 4:**
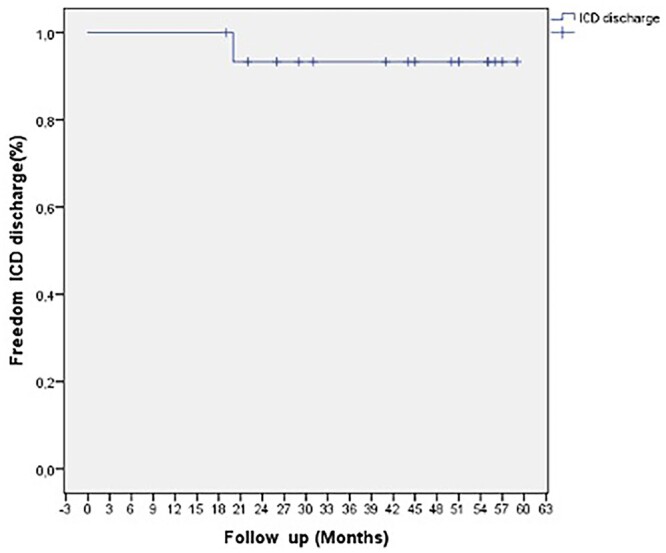
Kaplan–Meier curve showing 93.7% (95% confidence interval, 51.5–61.3) freedom from ICD discharge in patients who underwent septal myectomy after previous alcohol septal ablation. ICD: implantable cardioverter-defibrillator.

Eventually, 2 (12.5%) patients required pacemaker implantation due to complete AV-block. Kaplan–Meier estimates of survival at 3 years were 100% (95% confidence interval, 100–100) in the studied group. In-hospital and overall mortality after septal myectomy accounted 0%. No cardiovascular events such as sudden cardiac death, cardiac arrest or advanced heart failure were registered during 2-year follow-up. Freedom from reoperation accounted 100% (95% confidence interval, 100–100) at 3-year follow-up. During the same period, 1 (6.3%) patient had an ICD discharge occurred 1.8 years after surgery. Freedom from ICD discharge was 93.7% (95% confidence interval, 51.5–61.3; Fig. 4). Additional major complications after septal myectomy are listed in Table 3.

## DISCUSSION

The results of our study showed that surgical septal myectomy in patients with previously failed ASA are a safe and effective procedure. The surgical intervention allows adequately release the LVOT and manage pathophysiological manifestations of HOCM by resecting anomalous secondary chordae, mobilizing PM and plicating the AML. In addition, the rate of postoperative complications is similar to that in our larger septal myectomy experience [14]. However, this study has shown that these patients are at risk of postoperative AV-block due to already existing right bundle branch block after ASA.

ASA is a mini-invasive catheter-based method of HOCM treatment that involves the injection of ethanol into the septal branch of the left anterior descending artery, thus causing the local myocardial infarction. It results in a certain degree myocardial remodelling and release of the LVOT which contributes to reduction of SPG and relieving obstruction. Despite the attractive features of this procedure, there are still a number of characteristics that limit the routine applicability of ASA in patients with HOCM.

The anomalous anatomy of the MV subvalvular apparatus can be found in the vast majority of patients with HOCM and plays an important role in manifestation of the disease [10, 15]. The structures like anomalous secondary chordae, muscular bands or fibrotic attachments between the AML and PM, IVS or LV free wall can sometimes be observed and managed only intraoperatively and cannot be resolved by ASA. Moreover, several studies report that inconsistency of the septal artery blood supply and hypertrophic obstruction in the ablation target zone may cause recurrent or residual symptomatic LVOT obstruction post-ASA [16, 17]. Another factor that contributes to the SPG recurrence is collateral blood flow that supplies the ischaemic basal septum after ASA.

Recent research conducted by Q. Yang *et al.* reports that the pathologic features in the ablation zone with interstitial fibrosis, myocardial necrosis and transmural myocardial scaring post-ASA, makes the subsequent septal myectomy more challenging to some extent. Our study also defines the technical difficulties which the surgeon can be confronted by. We observed calcified IVS in 2 (12.5%) out of 16 patients and suggested a slight modification of the standard myectomy which provides optimal visual control during LVOT release. Moreover, our study demonstrates an importance of the preoperative CT/CMR planning in septal myectomy. This method was first described in the work of Spirito *et al.* [18] According to their study, in 112 consecutive HOCM patients, depth and length of the myectomy planned at CMR were compared with those of the septal muscle excised in a single piece in all patients. Thickness and length of the planned myectomy showed a significant correlation with the excised muscle (*R*^2^ = 0.345; *P* < 0.001; and *R*^2^ = 0.358; *P* < 0.001, respectively). Thus, preoperative planning provides high-resolution images of septal morphology and allows to perform a standardized and apically extended septal excision that was associated with favourable outcome. In our study, applying this approach made myectomy more accessible and as much extended as possible preventing from iatrogenic VSD.

Another important finding is that the presence of fibrosis on the basal septum after ASA is often associated with rhythm disturbances. In some reports, risk of complete heart block may reach 20% after ASA [19]. In our study, only 1 patient had post-ASA pacemaker implantation. However, 2 (12.5%) patients required pacemaker after surgery due to complete AV-block. The explanation for this is that septal myectomy commonly produces a left bundle branch block [5], and thus pre-existing right bundle branch block at the time of surgical myectomy may confer an increased risk of complete heart block. Therefore, clinicians utilizing ASA for the treatment of HOCM should be cautious of conduction system injury, and patient selection should be optimized.

Up to date, only several existing studies report the effectiveness and long-term outcomes of septal myectomy after previous ASA. The largest ones, conducted by E. Quintana *et al.* [5] and Q. Yang *et al.* [20], report about the increased rate of complications after surgery in post-ASA patients. Moreover, little is known regarding the risk of late arrhythmias in these patients. Although, a recent study of Balt *et al.* [21] showed that only 7% of patients experienced malignant tachyarrhythmia (ventricular tachycardia/ventricular fibrillation) in the first post-ASA month, while no ventricular tachycardia/ventricular fibrillation was observed later, no relative data concerning myectomy patients after failed ASA exist up to date. In our studied cohort, only 1 patient had ICD discharge 1.8 years after surgery. However, considering the small number of studied patients and relatively short follow-up period, the risk of late arrhythmias occurrence should be the object of further studies.

### Study limitations

This study had several limitations to mention. First, the study was a non-randomized retrospective cohort analysis with a small sample (16 patients). Therefore, the conclusions must be confirmed in a large-sample cohort study. Another limitation was the inability to define the rate of failure of ASA since the procedure was not performed at our Institute. Finally, the relatively short follow-up time does not demonstrate the full spectrum of perioperative outcomes and requires further analysis.

## CONCLUSION

Surgical treatment of HOCM in patients who previously underwent ASA requires surgical approach that will provide correction of all pathological manifestations of the disease. Routine preoperative CT or CMR provides detailed anatomy of the anomalous LV and MV structures as well as allows to measure the extension of myectomy preserving from iatrogenic VSD. When performing septal myectomy of calcified IVS, it is recommended to avoid applying 1-piece technique since fragmental myectomy allows visually control the adequacy of LVOT release.

## Notes

Presented at the 35th Annual Meeting of the European Association for Cardio-Thoracic Surgery, Barcelona, Spain, 13–16 October 2021.

## ABBREVIATIONS

AML: Anterior mitral leaflet
ASA: Alcohol septal ablation
AV: Aortic valve
CMR: Cardiac magnetic resonance
CT: Computed tomography
HOCM: Hypertrophic obstructive cardiomyopathy
ICD: Implantable cardioverter-defibrillator
IVS: Interventricular septum
LV: Left ventricle
LVOT: Left ventricular outflow tract
MR: Mitral regurgitation
MV: Mitral valve
PM: Papillary muscles
SAM: Systolic anterior motion
SPG: Systolic pressure gradient
TEE: Transesophageal echocardiography
VSD: Ventricular septal defect
